# Simulation-based training in emotional intelligence and self-esteem: enhancing effectiveness and wellbeing in healthcare

**DOI:** 10.3389/fpubh.2025.1667192

**Published:** 2025-11-13

**Authors:** María del Carmen Pérez-Fuentes, María del Mar Molero Jurado, Armando Romanos-Rodríguez, Ana B. Barragán Martín, Francisco J. Gómez-Gómez, Javier Aguado-Campos

**Affiliations:** 1 Department of Psychology, University of Almería, Almería, Spain; 2 IAVANTE, Andalusian Public Foundation Progress and Health-FPS, Sevilla, Spain

**Keywords:** emotional intelligence, self-esteem, health professionals, professional wellbeing, quality of patient care, health training, educational interventions, stress management

## Abstract

**Introduction:**

The study focuses on the training of health professionals, traditionally based on technical skills and specialized knowledge. However, the importance of integrating emotional intelligence and self-esteem has gained recognition for its impact on the quality of patient care and professional wellbeing. This study examines how targeted interventions in these areas can improve wellbeing and professional effectiveness in primary care settings.

**Method:**

A multicenter study was conducted with a pre-experimental design, assessing participants before and after the intervention, with no control group. Participants were 106 physicians and nurses in primary care centers in Andalusia, Spain. The intervention consisted of an Advanced Life Support (ALS) training program implemented in a blended learning format that combined online theoretical instruction with in-person practical sessions. The course integrated theoretical modules, hands-on workshops, and simulation-based exercises aligned with international resuscitation guidelines. The Brief Inventory of Emotional Intelligence and the Rosenberg Self-Esteem Scale were used. Data analysis was performed using nonparametric tests and the Wilcoxon test to assess pre- and post-intervention variations.

**Results:**

Correlations between self-esteem and several dimensions of emotional intelligence showed significant post-intervention increases. The intrapersonal and interpersonal factors of emotional intelligence showed significant improvements in their mean scores. However, no significant changes in stress management, mood, or self-esteem were observed in the total sample.

**Conclusion:**

The study highlights the efficacy of incorporating emotional intelligence and self-esteem training in the training of health professionals, demonstrating improvements in stress management and adaptability. The variations in the effects of the intervention suggest the need to adapt future interventions to the specific characteristics of each profession. Integrating these programs into health education could significantly enhance the quality of patient care and the wellbeing of health professionals.

## 1 Introduction

The training of health professionals has traditionally focused on the mastery of technical skills and specialized knowledge, essential components for an effective clinical practice (1). However, the relevance of incorporating personal aspects such as emotional intelligence, self-esteem and stress management has begun to be widely recognized (2), as well as the development of social skills (3). These qualities are not only vital for the wellbeing of healthcare professionals, but also contribute significantly to the quality of patient care, improve communication, and support ethical decision making in the face of complex dilemmas (4). By integrating these personal dimensions into the education of healthcare professionals, one aspires to develop more resilient, compassionate, and efficient individuals equipped to handle both the technical and human challenges presented by the healthcare field (5). Recent research highlights the importance of emotional intelligence in the prevention of mobbing among nursing staff, highlighting the mediating role of social support and sensitivity to anxiety (6) and explores the use of machine learning techniques to predict sensitivity to sensory processing in nursing students (7), emphasizing the need to address these aspects in the training of health professionals.

### 1.1 Emotional intelligence and its impact in the healthcare environment

Defined by Salovey and Mayer (8) as the ability to perceive, appraise, express and influence one’s own and others’ emotions, emotional intelligence is crucial in high-stress work environments, such as healthcare contexts. In professions of high emotional demand and constant stress, such as nursing and medicine, emotional intelligence not only contributes to better personal and professional management, but also improves the quality of patient care (9, 10), reducing work stress and emotional exhaustion, prevalent problems in this field (11). In this context, research by Ni’matuzahroh et al. (12) highlight the positive relationship between emotional intelligence and psychological wellbeing of healthcare workers, indicating that those with high emotional competencies experience lower levels of burnout and higher job satisfaction.

This positive relationship also occurs with critical aspects of health, such as empathy, self-efficacy and general wellbeing of healthcare professionals (13). Hence, several studies have shown that specific emotional intelligence training programs can have a significant impact on the mental health and professional effectiveness of healthcare personnel (14). For example, a direct relationship has been observed between emotional intelligence and the ability to resolve conflicts (15), perception of organizational climate and adherence to ethical principles in daily practice (16). When we talk about specific training programs we must take into account e-learning training programs, coaching training (17) when it comes to improving emotional intelligence, empathy skills, self-esteem, and also leadership development and time management programs. These programs can be customized to meet individual and group needs, focusing on active methodologies that promote participation and hands-on learning (18, 19).

### 1.2 Self-esteem: a critical factor in the wellbeing of health care professionals

Self-esteem, understood as the valuation a person has of him/herself, plays a crucial role in various aspects of life, including the professional sphere (20). In the case of health professionals, self-esteem not only impacts their psychological wellbeing, but also their ability to face the adversities and challenges inherent to their field of work (21). Hence, work stress in the healthcare setting can be affected by the presence of low self-esteem among professionals, affecting their professional performance and their state of personal wellbeing causing burnout. This phenomenon not only affects the mental and physical health of healthcare professionals, but can also compromise the quality of care provided to patients, underscoring the need to address self-esteem within the healthcare sector as a priority public health issue (22). Likewise, it has been observed that high self-esteem is associated with better coping skills, which translates into greater efficacy in handling difficult situations and more positive interactions with patients and among medical professionals (23). In other words, healthy self-esteem facilitates better stress management, contributes to greater job satisfaction and improves the quality of patient care (24, 25). Similarly, the moderating effect that self-esteem has on the relationship between burnout and the frequency of suicidal behaviors has been demonstrated (26), since the perception of self-efficacy enhances the ability to face challenges, while a solid self-esteem is fundamental for a person’s satisfactory social interaction and connection (13).

### 1.3 Relationship between emotional intelligence and self-esteem

In the healthcare setting, emotional management and strong self-esteem are recognized as key factors for effectively coping with high-pressure situations. Pérez-Fuentes et al. (27) highlighted the existence of a negative correlation between emotional intelligence and self-esteem with burnout in healthcare professionals, especially emphasizing the impact on emotional exhaustion within the nursing collective (28). This highlights the relevance of these personal qualities. However, traditionally, medical training has prioritized the development of technical skills, leaving emotional intelligence and self-esteem, which have a direct impact on work and personal performance, in the background (29).

Healthcare education and professional development have begun to recognize the need to incorporate the strengthening of self-esteem and emotional intelligence in their programs, understanding that these aspects are as crucial to patient care as technical skills (29). Recent research highlights that a holistic approach in healthcare training, including the development of emotional and personal competencies, contributes significantly to improving the quality of care, patient satisfaction and the wellbeing of healthcare workers (13, 30, 31).

It has been shown that specific programs that develop emotional intelligence can reinforce the self-esteem of healthcare personnel, increasing their resilience and ability to cope with work-related stress (2, 23). The relationship between emotional intelligence and self-esteem in these professionals is bidirectional: high emotional intelligence improves self-esteem by facilitating better emotional management, while positive self-esteem can boost the development of emotional and social skills. This link emphasizes the importance of addressing these constructs in an integrated manner in the training of healthcare personnel to promote both their wellbeing and the quality of patient care (29). For instance, a recent randomized clinical trial among primary health care nurses in Greece assessed changes in emotional intelligence following an educational intervention, demonstrating the feasibility and importance of such training in this specific setting (32).

In addition to the impacts observed in the healthcare sector, the relevance of fostering emotional intelligence and self-esteem to improve mental health, adaptation to change and extends to other professional settings (33, 34). Our study examines emotional intelligence and self-esteem in the context of primary care, evaluating the effect of an intervention aimed at strengthening these skills in physicians and nurses. Based on the literature and the study’s purpose, the following research questions were formulated: (1) Does participation in the intervention lead to significant improvements in emotional intelligence and self-esteem? (2) Are there differences in these effects between physicians and nurses? and (3) How are the dimensions of emotional intelligence related to self-esteem before and after the intervention?

Using a longitudinal pre-experimental design, we investigate how training interventions can enhance the psychological wellbeing and professional efficacy of healthcare workers. This design was selected because it enables the observation of changes in emotional intelligence and self-esteem over time within the same participants, thus providing robust evidence on the intervention’s effects. The study supports the hypothesis that higher levels of emotional intelligence and self-esteem contribute to improved patient care and greater wellbeing among healthcare professionals.

## 2 Materials and methods

This is a multicenter study with a pre-experimental design, where a pre- and post-intervention evaluation (without a control group), of a longitudinal nature, is applied.

### 2.1 Participants

The set of participants in this study included physicians and nurses active in primary care centers in Andalusia, Spain. The selection strategy adopted was non-random consecutive sampling, aimed at professionals enrolled in an Advanced Life Support training program. Inclusion criteria required participants to have completed their academic training in Medicine or Nursing, to be actively practicing within primary care centers belonging to the Andalusian Health Service, and to have fully completed the pre and post-training questionnaires. Exclusion criteria included having completed a similar Advanced Life Support course within the previous 2 years or failing to complete either evaluation phase. All participants who met these criteria successfully completed both assessments, and no cases were excluded due to incomplete data.

Regarding the demographic and professional characteristics of the subjects, there was variability in age, ranging from 21 to 65 years, which corresponds to the active working stage after completing their studies, with an average age of 44.71 years. The distribution by gender showed a female predominance of 62.26% in the total sample, with a notable participation in the areas of medicine and nursing.

With respect to professional experience, the overall average was 18.42 years. When analyzing the professions separately, nurses had more average experience, with 21.79 years, in contrast to physicians, whose average experience was 14.69 years.

The distribution of the sample by Andalusian provinces is detailed below: Almería was home to 17.92% (*n* = 19) of the participants, divided into 19.61% physicians and 16.36% nurses. In Cadiz, 20.75% (*n* = 22) of the sample was represented, with physicians making up 15.68% and nurses 25.45%. Granada had 21.70% (*n* = 23) of the participants, with a distribution of 23.53% physicians and 20.01% nurses. Seville had the highest proportion, with 39.63% (*n* = 42) of the total number of participants, of whom 42.18% were physicians and 38.18% nurses.

### 2.2 Instruments

Sociodemographic and occupational data were collected by means of an *ad hoc* questionnaire, which included questions on age, sex, education, profession, and length of service/experience in the profession.

As a measure of emotional intelligence, the Brief Emotional Intelligence Inventory (EQ-i-20 M) (35) is an adaptation for a Spanish adult population of the Emotional Intelligence Inventory: Young Version (EQ-i-YV) by Bar-On and Parker (36). It consists of 20 items with four response options on a Likert-type scale. It provides a score on five factors of emotional intelligence: Intrapersonal (e.g., “I can describe my feelings easily”), Interpersonal (e.g., “I understand well how other people feel”), Stress Management (e.g., “I find it difficult to control my anger”), Adaptability (e.g., “I can solve problems in different ways”), and Mood (e.g., “I feel confident”). In this case, the reliability indices obtained for the subscales, on each of the measures (pre-post), were as follows: Intrapersonal (PRE *ω* = 0.90, POST *ω* = 0.92), Interpersonal (PRE *ω* = 0.68, POST *ω* = 0.73), Stress Management (PRE *ω* = 0.81, POST *ω* = 0.84), Adaptability (PRE *ω* = 0.78, POST *ω* = 0.78), and Mood (PRE *ω* = 0.86, POST *ω* = 0.85).

The Rosenberg’s Self-Esteem Scale (37) was used to measure personal self-worth or self-esteem through a unidimensional structure, developed for use with both adolescents and adults. This instrument focuses on evaluating key aspects such as self-esteem and self-acceptance through 10 items, which are answered on a spectrum ranging from “strongly agree” to “strongly disagree.” For the scope of our research, the reliability obtained for this scale was *ω* = 0.78, both in the pre-test and post-test measures.

### 2.3 Procedure

The study followed a pre-experimental pretest-posttest design without a control group. The methodology implemented in this research is broken down as follows:

First, remote access to the training was made possible for users through an e-learning scheme. Then, before starting the training program, questionnaire booklets were distributed for completion (pre-test evaluation). The next phase consisted of the implementation of the “Advanced Life Support” training program, which consisted of 12 h divided into two sessions. At the end of the training, the booklets were handed out again to collect post-intervention data (post-test evaluation).

The “Advanced Life Support” program involved a total of 27 h of instruction, divided between 15 h of e-learning and 12 h of classroom practice, distributed over three afternoon sessions. The course methodology combined theory and practice in a blended format, using simulations to facilitate the acquisition of knowledge and skills necessary for the effective management of cardiorespiratory arrest (CRA), in accordance with the recommendations of the European Resuscitation Council.

The training content was organized in five thematic blocks, covering both knowledge competencies and practical skills, and was developed in two phases:

- In the e-learning phase, participants addressed the theoretical contents through the platform, using resources such as web content, multimedia (videos and presentations, PDF documents), activities and tasks for evaluation and self-evaluation, as well as a general forum to resolve doubts.
- The classroom phase allowed for the practical application of theoretical knowledge, focusing on technical and non-technical skills through hands-on workshops and simulations with feedback.

The teaching team was composed of seven professionals, including the course director, a physician with extensive experience in VAS and responsible for the training, nursing and medical instructors, and instructor candidates evaluated during the course. All of them had the necessary experience and pedagogical skills to ensure the quality of the training process, including knowledge in VAS and experience in clinical simulation.

The ALS course was given by IAVANTE with the collaboration of the Ministry of Health of the Andalusian Regional Government, the Spanish CPR Council and the European Resuscitation Council.

The research was approved by the Committee of Bioethics of the University of Almería with reference UALBIO2023/025 and the study adhered to the World Medical Association’s Code of Ethics (Declaration of Helsinki). The professionals targeted for this training were approached by the course director, who explained the study’s objectives. They were then asked to give their consent to participate. If the professionals chose not to be part of the research study, they could still engage in the training program without their data being collected, ensuring their educational experience remained unaffected.

### 2.4 Data analysis

When evaluating the normal distribution of the study variables, it was confirmed that the distribution of the data analyzed did not meet the normality assumption (*p* < 0.05 was obtained in the Kolmogorov–Smirnov test in all cases), so nonparametric tests were used. Analyses were performed with SPSS v24 (38), with a value of *p* < 0.05 being considered statistically significant.

To verify the association between the variables (emotional intelligence and self-esteem), the bivariate correlation matrix was estimated, specifically with Spearman’s rho correlation coefficients, taking into account the two moments of measurement (pre and post). According to the criteria established in the scientific literature, a Spearman’s rho correlation coefficient up to 0.30 is considered to indicate a weak correlation, a value between 0.30 and 0.50 denotes a moderate correlation, and a coefficient above 0.50 is interpreted as a strong correlation (39).

In order to evaluate the variations in emotional intelligence and self-esteem scores before and after participation in the program, the Wilcoxon signed-rank test was used. In addition, the effect size was estimated using the rank-biserial correlation measure (rrb), considering the following cut-off points: 0.10 small, 0.30 medium, and 0.50 large (40).

To examine the reliability of the instruments used for data collection, McDonald’s omega coefficient is estimated, following the proposal and indications of Ventura-León and Caycho (41).

## 3 Results

In line with the study objectives, the following results describe the effects of the intervention on emotional intelligence and self-esteem, as well as the relationships between their main dimensions.

### 3.1 Emotional intelligence and self-esteem: correlations

To determine whether the dimensions of emotional intelligence were related to self-esteem, a Spearman’s rho correlation analysis was carried out for each of the measures (pretest and posttest), the correlation coefficients and their statistical significance are presented in Table 1.

**Table 1:** Correlations between emotional intelligence and self-esteem.

Sample	Self-esteem	Intrapersonal	Interpersonal	Stress management	Adaptability	Mood
Total sample (*N* = 106)	Pre-test					
	Spearman’s rho	0.045	0.055	0.361***	0.346***	0.450***
	*p*-value	0.647	0.573	<0.001	<0.001	<0.001
	Post-test					
	Spearman’s rho	0.180	0.196*	0.234*	0.410***	0.622***
	*p*-value	0.065	0.044	0.016	<0.001	<0.001
Physicians (*n* = 51)	Pre-test					
	Spearman’s rho	0.002	−0.013	0.431**	0.220	0.295*
	*p*-value	0.987	0.927	0.002	0.120	0.036
	Post-test					
	Spearman’s rho	0.221	0.185	0.224	0.294*	0.585***
	*p*-value	0.119	0.193	0.114	0.036	<0.001
Nurses (*n* = 55)	Pre-test					
	Spearman’s rho	0.083	0.189	0.334*	0.525***	0.665***
	*p*-value	0.548	0.167	0.013	<0.001	<0.001
	Post-test					
	Spearman’s rho	0.156	0.189	0.227	0.498***	0.651***
	*p*-value	0.257	0.168	0.095	<0.001	<0.001

Pre-test and post-test correlations. Total sample (*N* = 106) and professional groups (physicians *n* = 51 and nurses *n* = 55).

* < 0.05, ** < 0.01, *** < 0.001.

The results obtained before the intervention, for the total sample of participants showed that self-esteem, presented correlations with mood (Spearman’s rho = 0.45, *p* < 0.001), stress management (Spearman’s rho = 0.36, *p* < 0.001) and adaptability (Spearman’s rho = 0.35, *p* < 0.001).

After the intervention, the correlation between self-esteem and mood increased markedly (Spearman’s rho = 0.62, *p* < 0.001). This increase suggests a strong association, highlighting that the intervention might have strengthened the relationship between emotional state and self-esteem. Along these lines, greater strength in the self-esteem-adaptability association was also observed in the post-intervention measure (Spearman’s rho = 0.41, *p* < 0.001), underscoring the importance of adaptability in self-appraisal of competencies in career performance.

On the other hand, the correlation between self-esteem and stress management revealed a decrease in the strength of this association (Spearman’s rho = 0.23, *p* < 0.05), reinforcing the idea that the intervention had an impact on the identification of possible limitations in situations that require immediate and precise response. Another noteworthy fact is that the association of self-esteem and the interpersonal factor of emotional intelligence (Spearman’s rho = 0.20, *p* < 0.05), becomes significant after the intervention, although with values close to the limit for statistical significance and a weak correlation strength. This suggests that, while there is a trend toward a positive association after the intervention, these relationships are less strong compared to adaptability and mood.

Attending to the profession, in the group of physicians (*n* = 51), before the intervention, the correlation between self-esteem and stress management (Spearman’s rho = 0.43, *p* < 0.01), turned out to be moderate. A positive correlation, although of weak strength, was also observed between self-esteem and mood (Spearman’s rho = 0.29, *p* < 0.05).

After the intervention, the correlation of self-esteem with mood experienced a notable increase (Spearman’s rho = 0.58, *p* < 0.001), suggesting that the intervention may have had a positive impact on this association. For its part, the correlation with adaptability began to be significant at the post-intervention measure (Spearman’s rho = 0.29, *p* < 0.05), although the strength of the correlation was weak, it emphasizes the sustained relationship between adaptability and self-esteem in the context of medical practice.

Finally, in the group of nurses (*n* = 55), before the intervention, the strongest correlations in self-esteem were observed with mood (Spearman’s rho = 0.66, *p* < 0.001) and adaptability (Spearman’s rho = 0.52, *p* < 0.001). The strength of this correlation suggests that emotional state and the ability to adapt to change have an impact on nurses’ appraisal of their professional and personal performance. A positive correlation was also identified in stress management (Spearman’s rho = 0.33, *p* < 0.05). This moderate correlation highlights the importance of effective stress management in nursing practice, underscoring how the ability to manage stress is linked to building self-esteem.

After the intervention, the correlation of self-esteem with mood remained strong (Spearman’s rho = 0.65, *p* < 0.001), as did the relationship with adaptability (Spearman’s rho = 0.50, *p* < 0.001). On the other hand, the correlation with stress management after the intervention was no longer significant, as was the case with this association in the pre-test.

### 3.2 Effects of the training program on emotional intelligence and self-esteem

In Table 2, an increase in the mean score of the intrapersonal factor of emotional intelligence can be observed with statistically significant results (W = 1033.00, z = −2.67, *p* < 0.01) and a biserial rank correlation of −0.35, indicating a moderate improvement. Similarly, for the interpersonal factor, a significant improvement was found (*W* = 829.00, *z* = −3.01, *p* < 0.01) and a biserial correlation of −0.40. In addition, adaptability showed a significant improvement (*W* = 838.50, *z* = −2.21, *p* < 0.05), even though the mean pre- and post-intervention scores were similar.

**Table 2:** Wilcoxon paired-sample test for emotional intelligence and self-esteem.

Intelligence E	Measures				Paired samples test			
	Pre-test		Post-test		*W*	*z*	Sig.	Rank-biserial Correlation
	*M*	SD	*M*	SD				
Intrapersonal	9.78	3.05	10.33	2.48	1033.00	−2.67**	0.007	−0.35
Interpersonal	11.69	1.81	12.13	1.55	829.00	−3.01**	0.002	−0.40
Stress management	12.83	2.34	13.02	2.02	1228.00	−1.22	0.222	−0.16
Adaptability	11.11	2.05	11.47	1.62	838.50	−2.21*	0.027	−0.31
Mood	11.85	2.19	12.09	1.93	989.50	−1.48	0.136	−0.20
Self-esteem	32.37	4.56	32.93	3.92	1901.50	−1.40	0.159	−0.17

Paired samples Wilcoxon’s test (*N* = 106).

*M*, mean; SD, standard deviation; *W*, Wilcoxon signed-rank test. * < 0.05, ** < 0.01.

On the other hand, no significant changes were reported in stress management, mood or self-esteem, where mean scores increased slightly, without reaching statistical significance.

Table 3 presents the results obtained after the analysis of pre-post intervention means in the subsample of physicians. In this case, it was self-esteem that presented a significant improvement (*W* = 370.50, *z* = −2.05, *p* < 0.05) and a biserial correlation of −0.34, indicating a moderate improvement.

**Table 3:** Wilcoxon paired-sample test for emotional intelligence and self-esteem in physicians.

Intelligence E	Measures				Paired samples test			
	Pre-test		Post-test		*W*	*z*	Sig.	Rank-biserial correlation
	*M*	SD	*M*	SD				
Intrapersonal	9.74	2.94	10.34	2.13	291.50	−1.80	0.069	−0.32
Interpersonal	11.92	1.95	12.12	1.60	265.00	−1.07	0.284	−0.20
Stress management	12.75	2.54	12.85	2.17	301.50	−0.49	0.624	−0.09
Adaptability	11.17	1.81	11.55	1.33	191.00	−1.60	0.110	−0.32
Mood	11.82	2.39	11.92	1.98	213.00	−0.10	0.930	−0.02
Self-esteem	31.38	4.45	32.50	3.57	370.50	−2.05*	0.040	−0.34

Paired samples Wilcoxon’s test (Doctors *n* = 51).

*M*, mean; SD, standard deviation; *W*, Wilcoxon signed-rank test. * < 0.05.

Table 4 shows the results obtained in the subsample of nurses, where changes in pre- and post-test scores were recorded, with some factors showing statistically significant improvements. Intrapersonal emotional intelligence experienced an increase in mean scores, reaching a trend statistical significance (*W* = 235.00, *z* = −1.96, *p* = 0.049) and a biserial rank correlation of −0.37, indicating a moderate improvement. Most notably, interpersonal intelligence showed significant improvement (*W* = 153.00, *z* = −3.15, *p* < 0.01) and a biserial correlation of −0.59, reflecting substantial improvement.

**Table 4:** Wilcoxon paired-sample test for emotional intelligence and self-esteem in nurses.

Intelligence E	Measures				Paired samples test			
	Pre-test		Post-test		*W*	z	Sig.	Rank-biserial correlation
	*M*	SD	*M*	SD				
Intrapersonal	9.80	3.18	10.31	2.79	235.00	−1.96*	0.049	−0.37
Interpersonal	11.48	1.66	12.13	1.51	153.00	−3.15**	0.002	−0.59
Stress management	12.90	2.16	13.18	1.87	323.00	−1.17	0.242	−0.21
Adaptability	11.06	2.26	11.40	1.86	236.00	−1.52	0.127	−0.29
Mood	11.88	2.00	12.25	1.89	294.50	−1.76	0.076	−0.32
Self-esteem	33.28	4.50	33.34	4.21	605.50	0.18	0.861	0.03

Paired samples Wilcoxon’s test (nurses *n* = 55).

*M*, mean; SD, Standard deviation; *W*, Wilcoxon signed-rank test. * < 0.05, ** < 0.01.

## 4 Discussion

The growing importance of emotional intelligence and self-esteem in the training and wellbeing of health professionals has been emphasized through various studies, such as those of Guerra-Báez (1), Pérez-Fuentes et al. (2), and Karimi et al. (4). These studies highlight how these personal aspects not only benefit the wellbeing of healthcare professionals but also improve the quality of patient care, facilitate effective communication, and promote ethical decisions in complex situations. Our results confirm and extend these findings, showing that after a focused intervention, significant improvements were observed in several dimensions of emotional intelligence, particularly in the intrapersonal and interpersonal areas. Similar results were reported by Fragkaki et al. (32) in a randomized clinical trial conducted with primary health care nurses, where an educational intervention also led to significant gains in emotional intelligence, supporting the effectiveness of such training programs in healthcare contexts. These improvements are directly associated with an increase in the professionals’ ability to effectively manage stress and adaptability, essential elements in the high-demand healthcare environment. Furthermore, self-esteem showed a positive correlation with these dimensions, indicating its critical role in the wellbeing and professional efficacy of healthcare workers, in line with previous research (21, 24).

Although the intervention did not produce significant changes in all dimensions of emotional intelligence (stress management and mood) or self-esteem in the total sample, it is important to note that significant improvements in self-esteem were observed among physicians, especially in relation to mood, as in the study by Molero et al. (23) where physicians with high self-esteem showed better stress management and mood. This suggests that the effects of such interventions may vary by profession and individual starting point in terms of emotional intelligence and self-esteem.

In contrast, the intervention in the nurses’ group had no significant changes in the relationship between self-esteem and mood and adaptability. However, the relationship with stress management ceased to be significant once the intervention was implemented. These results may be due to the emotional and physical overload and exhaustion experienced by nurses given their repeated exposure to the pain and suffering of their patients, who may sometimes be close to their circle (28).

These findings reinforce the idea that emotional intelligence and self-esteem are interconnected and fundamental elements in the healthcare context, contributing not only to the personal wellbeing of the professional but also to a more effective and humanized clinical practice (13, 30, 31). The positive correlation between emotional intelligence and self-esteem, particularly with regard to stress management and mood, suggests a virtuous cycle where improvements in one can foster improvements in the other.

Therefore, these results support the integration of emotional intelligence training and the development of self-esteem in health education curricula (1), as a key strategy to improve not only the mental health and wellbeing of health professionals but also the quality of care provided to patients (29, 30). Hence, the relevance of adopting a holistic approach in the education of health professionals, which contemplates the development of technical as well as emotional and personal skills (9, 10).

### 4.1 Limitations

Future research should explore specific interventions designed for different roles within the healthcare setting and assess their long-term impact on professional wellbeing and quality of care. Which could offer a more generic view to customize training programs. In addition, there is a clear need to study the long-term impacts of these interventions on variables such as psychological wellbeing, job satisfaction and quality of patient care, using longitudinal designs that allow a deeper understanding of their sustained effects over time.

The study has limitations that should be considered. The pre-experimental design, with no control group, limits the ability to attribute direct causality to the interventions performed, and also, the brevity of the post-intervention follow-up prevents assessment of the persistence of long-term effects. These limitations, far from detracting from the value of the study, offer crucial opportunities for future research.

### 4.2 Practical implications

These findings provide important contributions to decision-making in the healthcare field. In particular, they offer a strong foundation to guide policymakers and healthcare administrators on the need to integrate emotional intelligence and self-esteem into training and professional development programs. Embedding such competencies within the strategic planning of healthcare services may help reduce professional burnout, improve organizational climate, and, consequently, enhance the quality of care.

Furthermore, the intervention described demonstrates promising potential for replication across different contexts and healthcare systems, including those with diverse primary care structures or more limited resources. Although cultural and organizational adaptations will be required, our results suggest that promoting emotional skills can serve as a cross-cutting strategy to strengthen professionals’ resilience and the quality of care.

Finally, the findings also have direct implications for undergraduate health sciences education. Rather than being restricted to continuing education, these competencies should be integrated into university curricula, supporting a more comprehensive approach that includes both technical and emotional skills from the earliest stages of training. Such an approach can better prepare future professionals, equipping them with greater adaptability, communication, and stress management abilities, qualities essential for effective clinical practice.

## 5 Conclusion

This research highlights the importance of strengthening emotional intelligence and self-esteem for the wellbeing and performance of health professionals. Through an intervention, significant improvements were observed in the dimensions of emotional intelligence, highlighting the intrapersonal and interpersonal areas, along with a positive correlation between emotional intelligence and self-esteem. The results suggest that enhancing these abilities may improve stress management and adaptability in demanding healthcare environments. The research reveals variations in the effects of the intervention among different professional groups, pointing to the need to tailor future interventions to the specific characteristics of each role within the healthcare sector. Integrating emotional intelligence and self-esteem training into educational programs for healthcare professionals not only benefits their mental health and wellbeing, but also enhances the quality of patient care. This study provides evidence on the value of developing emotional and personal competencies in health education, advocating for a holistic educational approach that prepares professionals to effectively meet today’s health care challenges.

The practical implications of this work suggest that the integration of emotional intelligence and self-esteem programs within health education enhances the adaptability and stress management of professionals, thus improving patient care. Adapting these interventions for different professional roles and their inclusion in continuous development programs promotes a more comprehensive and effective healthcare practice, effectively facing the challenges of today’s healthcare.

## Data Availability

The raw data supporting the conclusions of this article will be made available by the authors, without undue reservation, upon reasonable request.
